# Can blood glucose value really be referred to as a metabolic parameter?

**DOI:** 10.1007/s11154-019-09504-0

**Published:** 2019-05-15

**Authors:** Kornél Simon, István Wittmann

**Affiliations:** 1 County Hospital Department of Internal Medicine, Siófok, Hungary; 2 2nd Department of Medicine and Nephrological Center, Faculty of Medicine, University of Pécs, Pécs, Hungary

**Keywords:** Blood glucose, Euglycaemia, Eumetabolism, Dysmetabolism, Diabetes, Chronic stress

## Abstract

In clinical guidelines, near-normoglycaemia is recommended as the basic therapeutic target in diabetes mellitus. This proposal suggests that euglycaemia is associated with eumetabolism and that hyperglycaemia is an indicator of dysmetabolism. The authors analysed the relationship between short/long-term blood glucose values and cellular metabolism in various pathophysiological settings. The following types of dysmetabolism are suggested: “hyperglycaemic dysmetabolism based on insulin deficiency”, “hyperglycaemic dysmetabolism based on glucose toxicity”, “euglycaemic dysmetabolism”, “dysmetabolism of ischaemic/reperfusional origin”, and “chronic stress-mediated dysmetabolism”. The relationship between dysmetabolic states of various origin was also analysed. The authors conclude that the blood glucose value can only be accepted as a general metabolic parameter with marked limitations. The main arguments of this statement are that euglycaemia is not necessarily associated with eumetabolism and that acute hyperglycaemia does not necessarily indicate dysmetabolism. Identical cell metabolic performance can be supported by different biochemical energy-producing mechanisms associated with identical blood glucose values. Both positive and negative metabolic balance of cell metabolism can occur at identical blood glucose values. A further finding is that chronic hyperglycaemia acts simultaneously as a marker and as a maker of dysmetabolism; therefore, the achievement of near normoglycaemia remains the basic therapeutic goal in diabetes treatment. Insulin administration can beneficially influence dysmetabolic states of various origins. In the evolution of and interrelationships among various dysmetabolic states, the central role of chronic stress is emphasized. Discrepancies between blood glucose values and cellular metabolism are substantiated by the transporter nature of the blood glucose value; this value reflects the result of bidirectional glucose movement into and out of the tissues.

## Introduction

The term “cell metabolism” refers to all the events involved in energy-providing processes of the cell. In our terminology, eumetabolism defines a normal metabolic state in which the energy balance of the cell is positive, the resting creatine phosphate/adenosine triphosphate ratio is normal, and the magnitude of the inducible reserve metabolic capacity is also normal.

The term “dysmetabolism” defines a pathologic metabolic state in which the energy balance of the cell is negative, the creatine phosphate/adenosine triphosphate ratio is subnormal, and the magnitude of the inducible reserve metabolic capacity is diminished.

The need to define the metabolic state is justified by the fact that the common denominator of all pathological clinical settings can be characterized by the obligatory presence of abnormal cell metabolism (dysmetabolism). The difference between various pathologic states is, on the one hand, the presence of dissimilar causal factors (physical, chemical or biological cause) leading to dysmetabolism; on the other hand, the difference is related to the diverse organic localization of cell dysmetabolism.

The focus of our review is on diabetes mellitus because diabetes is considered the most common metabolic disorder; hyperglycaemia is its consistent marker, and the positive association between severity of hyperglycaemia and that of metabolic disorder (dysmetabolism) is a fundamental thesis.

It is well known that according to professional guidelines for treating chronic heart failure and acute coronary syndromes [1], the “near normoglycaemic (euglycaemic) state” is declared as the diabetes-specific therapeutic goal.

Based on the foregoing, some questions can be raised (Table 1). To answer these questions, we studied the relationship between blood glucose values and metabolic states in various pathological conditions.

**Table 1:** Main questions

Does euglycaemia really indicate eumetabolism?	
Does hyperglycaemia really indicate dysmetabolism?	
Does insulin treatment really beneficially influence dysmetabolism?	

## The different nature of metabolic disorder (dysmetabolism) in type 1 *vs*. type 2 diabetes

The relationship between blood glucose and cell metabolism of type 1 diabetics shows a sharp contrast to that of patients with type 2 diabetes. The basic pathophysiological differences between the two forms of diabetes are summarized as follows.

In type 1 diabetes, the aetiopathological factor is insulin deficiency. On the one hand, insulin is required for glucose uptake by cells in insulin-dependent tissues. On the other hand, in all human cells, the translocation of glucose-transporter carrier-protein molecules from the intracellular space to the cellular membrane is governed by insulin. When insulin shortage occurs, the activity of this process is disturbed, and the rate of glucose uptake decreases, resulting in hyperglycaemia in the extracellular space. The more severe the insulin deficiency is, the more highly expressed the glucose uptake disturbance is, and the greater is the resultant hyperglycaemia. Therefore, hyperglycaemia can be defined as a “marker” of insulin deficiency.

As a consequence of insulin deficiency, the translocation of insulin-insulin receptor units from the cell membrane to the cytosol (internalization and endocytosis of insulin) is also decreased; this decreases the effect of insulin on the enzymes of the Krebs (citric acid) cycle, resulting in general cellular metabolic disorder (dysmetabolism) of the whole organism.

Dysfunction of mitochondria caused by insulin deficiency characteristically leads to susceptibility to the generation of ketone bodies. The more pronounced the insulin deficiency is, the greater are the metabolic disorder and the magnitude of ketosis.

In metabolic disorder of type 1 diabetics, which is defined as “hyperglycaemic dysmetabolism based on insulin deficiency”, both hyperglycaemia and ketosis can be viewed as markers of insulin deficiency.

Insulin administration effectively controls both hyperglycaemia and ketosis. The more pronounced the hyperglycaemia and the ketosis are, the greater is the amount of insulin required for normalization.

The metabolic state of patients with metabolic syndrome and of type 2 diabetics characterized by insulin resistance and resultant hyperinsulinaemia differs from that of non-diabetics and patients with type 1 diabetes. It is important to emphasize that this metabolic disorder can develop even in the normoglycaemic state; therefore, this type of dysmetabolism is referred to as “euglycaemic dysmetabolism”.

It can be suggested that the functioning of signal transduction systems is altered in both metabolic syndrome and type 2 diabetes [2]. The reduced availability of glucose transporters and the resulting diminished cellular glucose uptake could result in insulin hypofunction. Thereby, subnormal inducible metabolic reserve capacity could theoretically be expected in some tissues and organs. This assumption is supported by the results of myocardial functional tests performed in patients with “euglycaemic dysmetabolism” [3, 4].

In patients with metabolic syndrome and concomitant euglycaemia, decreased speed of peak diastolic flow through the mitral valve and concomitant increased atrial pressure can be documented by echocardiography [5]. The speed of the mitral annulus movement during systole and the excursion amplitude of the mitral valve are similarly decreased in patients with metabolic syndrome [6]. It has also been observed that the compensatory hyperkinesis of the periinfarct zone of “mixed” diabetic patients is diminished compared to that of non-diabetics [7]. This means that the inducible metabolic reserve and contractile capacity of diabetic patients are lower than those of healthy subjects.

The one-month survival of diabetic patients with acute myocardial infarction is worse than that of non-diabetics, although the anatomical characteristics of coronary arteriography and the prevalence of cardiovascular serum risk factors are identical in both groups of patients [7]. This indicates that the disordered cardiac metabolic state of diabetic patients (i.e., the diabetic cardiomyopathy) acts as an independent risk factor. The diabetic cardiomyopathy per se has a deteriorating effect on both diastolic and systolic function, resulting in a predisposition to the development of heart failure [8].

It is important to note that in patients with metabolic syndrome and type 2 diabetes, insulin resistance cannot be demonstrated in every cell in all organs. This means that in some tissues, the compensatory hyperinsulinaemia will lead to hyperfunctional activity. For example, hyperfunction of the tyrosine kinase receptor results in overproduction of various growth factors, which could play a role in the pathogenesis of micro- and macroangiopathy and left ventricular hypertrophy and in the augmented incidence of malignancies of diabetics. The increased renal sodium reabsorption, the augmented hepatic very low-density lipoprotein synthesis, the hyperactivity of hydroxymethylglutaryl coenzyme A reductase, the increased platelet adhesion and aggregation activity, and the central obesity found in patients with metabolic syndrome and in type 2 diabetics can also be viewed as examples of augmented insulin action.

These data demonstrate that in patients with metabolic syndrome and type 2 diabetes, who can be characterized by the term “euglycaemic dysmetabolism”, insulin-dependent functional activity (irrespective of hyperinsulinaemia) could be diverse or even inverse in different organs. It is important to note that the actual blood glucose value does not reveal this heterogeneity. This means that the blood glucose value provides no strict information about the actual metabolic state of various organs.

Therefore, the “euglycaemic dysmetabolism” of patients with metabolic syndrome and of type 2 diabetics fundamentally differs from the “hyperglycaemic dysmetabolism based on insulin deficiency” of type 1 diabetics; furthermore, the levels of blood glucose (euglycaemia *vs*. hyperglycaemia) could also be different.

## Breakthrough phenomenon

The concept of glucotoxicity includes the idea that hyperglycaemia generates additional hyperglycaemia. The pathophysiology of this phenomenon involves several mechanisms [2, 9]. This type of metabolic disorder could be referred to as “hyperglycaemic dysmetabolism associated with glucotoxicity”.

**Fig. 1:**
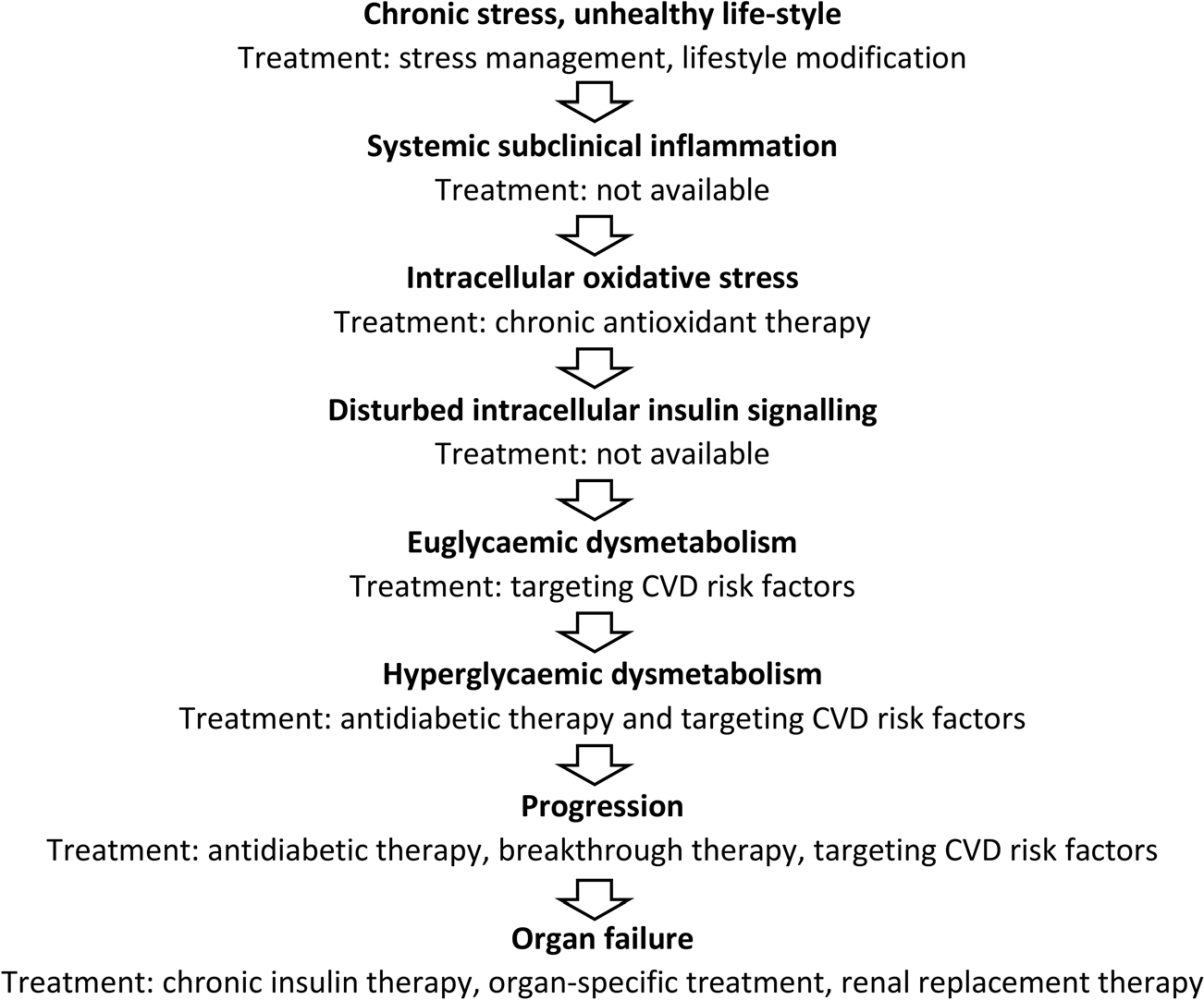
Pathogenesis of diabetes-related dysmetabolism

It is also well known that the significant reduction of blood glucose values achieved by administration of higher doses of insulin might “break through” the glucotoxicity; i.e., post-breakthrough lower blood glucose values can be maintained by lower insulin dosages or without insulin [2]. This means that “hyperglycaemic dysmetabolism based on glucotoxicity” can be alleviated by insulin treatment, probably as a result of the decrease in cellular oxidative stress and related subclinical inflammation due to lower production of cytokines (Fig. 1). This beneficial effect is temporary, although it may persist for some months or years.

In the mechanism of glucotoxicity, hyperglycaemia is not a “marker” but a “maker”; i.e., in this setting it not only indicates the disorder but even generates it. As opposed to “hyperglycaemic dysmetabolism based on insulin deficiency” and “euglycaemic dysmetabolism”, “hyperglycaemic dysmetabolism caused by glucotoxicity” is a metabolic disorder that has quite different underlying mechanisms.

It can be suggested that there is an interaction between the “maker” and the “marker” function of these hyperglycaemic states. On one hand, hyperglycaemia works as a “maker” by further aggravating the pre-existing hyperglycaemia caused by any mechanism. For example, in type 1 diabetes, “hyperglycaemic dysmetabolism caused by insulin deficiency” is further worsened by “hyperglycaemic dysmetabolism caused by glucotoxicity”. On the other hand, the magnitude of hyperglycaemia of any origin is a “marker” of the severity of the actual metabolic disorder regardless of that disorder’s origin. This means that hyperglycaemia works as a “marker” and as a ‘maker” at the same time.

The cardiovascular risks associated with “euglycaemic dysmetabolism” *vs*. “hyperglycaemic dysmetabolism based on glucotoxicity” were demonstrated in the Saint Antonio Heart Study data [10]. This investigation revealed that both “euglycaemic dysmetabolism” alone (patients with metabolic syndrome) and “hyperglycaemic dysmetabolism caused by glucotoxicity” alone (type 2 diabetics) independently increase cardiovascular risk, whilst the co-occurrence of the two risk factors leads to a significantly higher probability of cardiovascular events.

## Relationship between the microcirculation and tissue metabolism

There is a harmonic interrelationship between the microcirculation and tissue metabolism. This means, on one hand, that various transmitters that affect the microcirculation and cell metabolism act in a synergistic way. On the other hand, it is also well documented that both the microcirculation and the actual cell metabolism mutually influence each other’s functional state in the same direction. For example, in case of acute negative energy balance of the myocardium, adenosine generation is increased by degradation of the adenosine triphosphate pool. The resulting intracellular adenosine accumulation acts beneficially on the microcirculation and on the cellular metabolism as well in the following ways: first, adenosine-1 receptor activation downregulates the mostly energy-dependent function of the myocardium, i.e., its contraction; second, adenosine-2 receptor activation augments the microcirculatory flow; third, adenosine-3 receptors supposedly improve the metabolic performance of cellular metabolism [11].

It is well documented that in cases of coronary artery stenosis the index macrocirculation develops an adaptation that is triggered by the circumscribed repeated myocardial metabolic energy deficiency of ischaemic origin. The more significant the stenosis is and the more frequent and pronounced the resulting exercise-induced ischaemic attacks are, the more marked is the compensatory generation of new vessels (i.e., collateral development) in the macrovascular segment.

Similarly, it can be proposed that the damage to cells caused by diabetic metabolic disorder also induces a compensatory functional and anatomical adaptation of the microcirculation that results in the development of microangiopathy. This hypothesis is supported by the fact that microangiopathy is most frequent in patients in the pathologic state of diabetes that is characterized by the most marked metabolic disturbance. Furthermore, diabetic microangiopathy develops most significantly in organs with the highest metabolic activity (nervous system, kidney, and retina) and is presumably associated with the most frequent and most marked episodes of energetic deficiency [6, 12]. For example, the metabolic balance of the retina can only be provided by the maximal simultaneous activity of anaerobic glycolysis and aerobic oxidation [13].

Therefore, it is suggested that parenchymal metabolic disturbance should be viewed as the primary cause of the development of microangiopathy; i.e., retinopathy is not a secondary consequence of microangiopathy but rather its primary cause. However, over time, the secondary compensatory adaptation of the microcirculation turns into maladaptation: the markedly and permanently increased cardiovascular risk factors associated with diabetes gradually lead to endothelial dysfunction [3, 12], which results in perfusional disturbances. This means that a secondary metabolic disorder of ischaemic origin is added to the primary diabetic one. A vicious circle is developing; not only does the diabetic dysmetabolism induce microangiopathy, but microangiopathy also contributes to the development of additional dysmetabolism [8, 14].

It has been documented that the more severe the metabolic disorder caused by insulin deficiency is, the more marked is the resulting microangiopathy. It is also well-known that insulin therapy not only beneficially influences hyperglycaemia and dysmetabolism but can also reverse microangiopathy [8, 15].

## Relationship between macroangiopathy and hyperglycaemia

It is well documented that there is a strong relationship between diabetes and accelerated macroangiopathy. The association between diabetes and hyperglycaemia is also obvious. These statements raise the following question: what is the causal role of hyperglycaemia in the development of macroangiopathy in type 1 and type 2 diabetes, respectively?

It is known that in type 1 diabetes hyperglycaemia can be present for a long time, even for decades, without significant development of macroangiopathy [16, 17]. The macrovascular complications of type 1 diabetes are generally manifested in association with the development of renal complications. These data indicate that the presence of hyperglycaemia does not play a key pathogenetic role in the development of macroangiopathy in type 1 diabetes.

In type 2 diabetes, however, significant macroangiopathy develops early in the prediabetic phase, i.e., prior to the occurrence of hyperglycaemia and during the euglycaemic phase of metabolic syndrome [18, 19]. In this clinical setting, the evolution of macroangiopathy is also coincident with the presence of hypertension, dyslipidaemia and microalbuminuria [20], i.e., the development of macroangiopathy in type 2 diabetes is also not bound to the presence of hyperglycaemia.

The glucose paradox phenomenon strengthens the same conclusion: long-term control of blood glucose levels does not result in regression of macroangiopathy [15–17, 21]. In contrast, the “non-glucose non-paradox” clearly shows that in the medium term, effective treatment of hypertension, dyslipidaemia and platelet hyperactivity leads to significant improvement of macroangiopathy [22, 23].

All of these findings mean that neither “hyperglycaemic dysmetabolism based on insulin deficiency” nor “hyperglycaemic dysmetabolism caused by glucotoxicity” plays a decisive causal role in the pathogenesis of macroangiopathy in type 1 or type 2 diabetics.

## Do normal blood glucose values really indicate normal metabolism?

The fundamental feature of diabetes pathology is that the high blood glucose levels found in this condition cannot be spontaneously normalized. It is well documented that the hypoglycaemic susceptibility caused by administration of exogenous insulin can be successfully counteracted (up to a certain limit) by mobilization of endogenous glucose induced by catecholamines, glucagon, glucocorticoids, growth hormone, and thyroid hormone, resulting in movement towards normoglycaemia. The different magnitudes of endogenous glucose mobilization on the one hand and of tissue glucose uptake on the other hand may be associated both with different counter-regulatory hormonal activities and with significantly different metabolic activities of tissues at the same “normal” blood glucose level.

Furthermore, the specific effects of various hormones involved in this counter-regulation and in the provision of biochemical energy to cells are also well documented [24]. This means that the same euglycaemic state can disguise very different counter-regulatory hormonal activities and significantly different biochemical states of cellular metabolism that are not reflected by the actual blood glucose values.

It is well known from the results of euglycaemic clamp studies [25] that the simultaneous administration of various amounts of intravenous glucose and appropriate insulin dosages results in permanently normal blood glucose values. It is obvious that the identical blood glucose values observed during the euglycaemic clamp do not reveal either the significantly various degrees of glucose disposal or the very different metabolic activities of tissues.

## Metabolic promoters

The UK Prospective Diabetes Study Group demonstrated [26, 27] that although both metformin and insulin therapies similarly improved blood glucose levels in patients with type 2 diabetes, patients who were treated with metformin benefited more in terms of clinical outcome than those on insulin treatment. These data indicate the favourable metabolic effect of metformin, which seems to be independent of blood glucose levels. Metformin is believed to activate the so-called metabolic sensor (adenosine monophosphate-activated protein kinase), which is thought to improve cellular metabolic performance [28].

Several enzymes are involved in biochemical cellular energy processes, and all of them can theoretically be separately influenced. For example, in the clinical setting, dichloroacetate is used to increase the activity of the pyruvate dehydrogenase enzyme to decrease high serum lactic acid levels.

Dichloroacetate, trimetazidine, thiazolidindiones, statins, fibric acid, etomoxir, perhexilen, ranolazin, metformin, acarbose, glucagon-like peptide-1 analogs, nicorandil and other compounds have been proposed as metabolic modulators [29–31]. It should be noted that insulin can be considered the most potent metabolic promoter due to its complex effects on both cellular glucose uptake and the enzymes of the tricarboxylic (citric) acid cycle.

All metabolic promoters are thought to improve cellular metabolism in a “blood glucose-independent way”; i.e., the actual blood glucose value does not reveal the heterogeneity in actual cellular metabolism.

## Relationship between dysmetabolism of ischaemic/reperfusion origin, hyperglycaemia, and insulin therapy

It is well known that the long-term survival of infarct patients with stress hyperglycaemia at admission is significantly worse compared than that of those without stress hyperglycaemia [17]. This means that the ischaemic and reperfusional myocardial damage associated with the infarct is augmented by the metabolic disturbance related to acute hyperglycaemia. This additional myocardial damage could be manifested in the final infarct size and in the resultant survival time.

Similarly, it has also been shown that the survival period of patients who underwent percutaneous coronary intervention decreased in a manner that was proportional to the extent of hyperglycaemia detected during the intervention [32]. This also suggests that the higher the blood glucose value is, the greater is the extent of metabolic disturbance and that the metabolic disturbance adds to the ischaemic/reperfusional damage associated with percutaneous coronary intervention.

The administration of glucose insulin potassium solution to non-diabetic patients during bypass surgery significantly improved survival [33], presumably indicating that insulin therapy can significantly improve ischaemia/reperfusion-induced metabolic disturbances of the myocardium. Thus, insulin therapy can be beneficial even in a metabolic disorder of non-diabetic origin.

In the CREATE ECLA Trial group study [34], administration of glucose insulin potassium solution during the acute phase of myocardial infarct did not result in improvement of the survival rate of patients subjected to thrombolysis. Theoretically, the glucose insulin potassium solution can only be effective in patients with acute coronary artery occlusion if two requirements are met. First, the myocardial damage of the myocardial area at risk should be in a reversible, salvageable phase (i.e., treatment with glucose insulin potassium solution should be initiated rapidly after symptom onset). Second, the occluded coronary artery should be opened because without reperfusion irreversible damage, i.e., the evolution of necrosis, cannot be avoided. The beneficial effect of glucose insulin potassium solution depends on its ability to act as a metabolic promoter; it can prolong the duration of the reversible stage of injury and thereby postpone the onset of necrosis. Qui habet tempus, habet vitam (who has more time, wins life).

When patients in the CREATE ECLA Trial group study who received the glucose insulin potassium solution within the first hour of symptom onset and in whom the subsequent thrombolysis resulted in reperfusion were analysed, the administration of glucose insulin potassium solution was found to be associated with highly successful prolongation of survival compared to patients without these prerequisites [35].

The foregoing data indicate that insulin therapy (glucose insulin potassium solution) can significantly diminish myocardial “dysmetabolism of ischaemic and reperfusional origin”. This beneficial promotion of myocardial metabolism is not reflected in the concomitant blood glucose values.

## Relationship between stress, blood sugar values, and cellular metabolism

Stress reaction, as described by Selye [36], is a nonspecific complex mechanism of the whole organism that is aimed to eliminate any harmful agents (acute stress: fight or flight reaction) or to diminish the effect of any chronic deleterious action (chronic stress).

In acute stress, the general response of the heart can be characterized as follows [3, 37, 38]: tissue perfusion increases approximately tenfold, and a healthy man is able to augment the heart rate and the stroke volume threefold. Tissue respiration, i.e., myocardial oxygen uptake, is also doubled: the arteriovenous oxygen difference increases twofold. These alterations result in an approximately twenty-fold increment in the myocardial oxygen supply. It is reasonable to suppose that in the adaptive sequence of tissue perfusion, tissue respiration, and tissue metabolism not only the first (tissue perfusion) and the second (tissue respiration) components but even the third component (tissue metabolism) should also have some inducible reserve capacity [14, 21]. The clinical significance of the latter mechanism has not been investigated in detail. It has been documented that in acute stress the cardiac metabolism switches from free fatty acid burning to glucose consumption. The reason for this phenomenon is based on the fact that the rate-limiting factor in the energy-providing biochemical chain is access to oxygen. Since both glucose and free fatty acid metabolism result in CO_2_ and H_2_O end products and the oxygen content of the glucose molecule is significantly higher than that of free fatty acid molecules, less exogenous oxygen is needed to produce the same amount of adenosine triphosphate during glucose burning than during free fatty acid metabolism. The adenosine monophosphate-activated protein kinase could also have an influence on the efficacy of cellular metabolism [28].

The acute stress state can be characterized by normal or elevated blood glucose values. This means that the increased metabolic activity is not reflected by the simultaneous frequently normal blood glucose measurements, i.e., there is no mandatory association between the actual blood glucose value and the metabolic state. The acute stress-related, occasionally augmented blood glucose values associated with significantly increased metabolic performance cannot be viewed as a marker of the pathological metabolic state. This means that hyperglycaemia does not necessarily indicate dysmetabolism.

Under conditions of repeated stress perfusion, the respiration and metabolism of tissues are improved by additional reserve mechanisms, resulting in greater myocardial contractile performance [23, 38]. This adaptation is reflected by both increased mitochondrial number and development of left ventricular hypertrophy. None of these significant metabolic, functional, and morphological modifications are revealed by the concomitant blood glucose values.

In permanent (chronic) stress, increased long-term catecholamine release leads to the development of insulin resistance and consequent hyperinsulinaemia. All of these alterations will gradually result in predisposition to hyperglycaemia and diabetes [6].

Catecholamine mobilization significantly increases the activity of carnitine acyl transferase, leading to overflooding of the mitochondria with free fatty acids and excessive beta oxidation. Hyperactivity of carnitine acyl transferase inhibits the activity of pyruvic acid dehydrogenase, which in turn increases the likelihood of cytosolic lactic acid accumulation. According to insulin resistance, the glucose uptake of cells is also disturbed, resulting in a decrease in the supply of pyruvic acid derived from glucose, which in turn leads to insufficient synthesis of oxalate-acetic acid generation from the condensation of pyruvic acid and carbon dioxide [39]. The shortage of oxalate-acetic acid results in disturbed function of the tricyclic acid cycle, which leads to deficient macroerg phosphate generation and a tendency to ketosis development. As a consequence of ineffective free fatty acid burning, toxic fatty acid products accumulate, and the augmented activity of malonyl coenzyme-A increases intracellular triglyceride synthesis, resulting in lipotoxicity [40]. In this state, mitochondrial macroerg phosphate production is markedly diminished, and cytosolic anaerobic glycolysis, which is less efficient, becomes the main energy-producing mechanism [41, 42]. Glycogen accumulation by cells is augmented, and their inducible metabolic reserve capacity is decreased. This disordered metabolic state can be referred to as “chronic stress-mediated dysmetabolism”, i.e., metabolic remodelling [43, 44].

It should be emphasized that although chronic stress is characterized by elevated blood glucose values, this hyperglycaemia is not associated with augmented metabolic performance but rather with seriously impaired metabolic performance, i.e., there is no strict relationship between actual metabolic activity and blood glucose values.

The metabolic biochemical machinery of striated muscles in sprinters shows fundamental differences compared to that of marathon runners. The 100-m distance run by a sprinter is traversed using a single breath; the required energy is provided by the breakdown of macroerg phosphate reserves and by newly synthetized adenosine triphosphate molecules originating from anaerobic glycolysis; at the same time, a significant amount of oxygen debt is generated. The marathon runner develops no oxygen debt; i.e., his or her metabolism is characterized by a balanced steady state in which the main energy-providing biochemical mechanism is the burning of free fatty acids through the Krebs cycle. The blood sugar values of both marathon runners and sprinters values are normal or elevated and do not reflect the basically different biochemical mechanisms involved in their actual metabolic processes.

## Types of metabolic disorders of different aetiology

Pathological metabolism (dysmetabolism) can be triggered by various causes: hyperglycaemic dysmetabolism may be caused by insulin deficiency, and hyperglycaemic dysmetabolism may be caused by glucotoxicity, euglycaemic dysmetabolism, toxic dysmetabolism (e.g., sepsis), dysmetabolism of ischaemic/reperfusional origin, and chronic stress-mediated dysmetabolism (Table 2).

**Table 2:** Types of metabolic disorders

Hyperglycaemic dysmetabolism caused by insulin deficiency	
Hyperglycaemic dysmetabolism caused by glucose toxicity	
Euglycaemic dysmetabolism	
Toxic dysmetabolism (e.g., sepsis)	
Dysmetabolism of ischaemic/reperfusional origin	
Chronic stress-mediated dysmetabolism	

Chronic stress inevitably leads to a cascade of pathological consequences [26, 45]; these may include the evolution of insulin resistance, secondary hyperinsulinaemia, a propensity toward hyperglycaemia, and the development of diabetes [3, 15, 16, 46].

Permanently elevated serum levels of cardiovascular risk factors (haemodynamic, inflammatory, lipidaemic, haemostaseologic, and metabolic factors) associated with chronic stress lead to the development of macroangiopathy and microangiopathy and consequential deterioration of tissue perfusion, finally resulting in tissue dysmetabolism of ischaemic origin [14, 26, 47].

In addition, chronic stress is associated with the development of stress-mediated dysmetabolism (metabolic remodelling) [12, 14, 21].

**Fig. 2:**
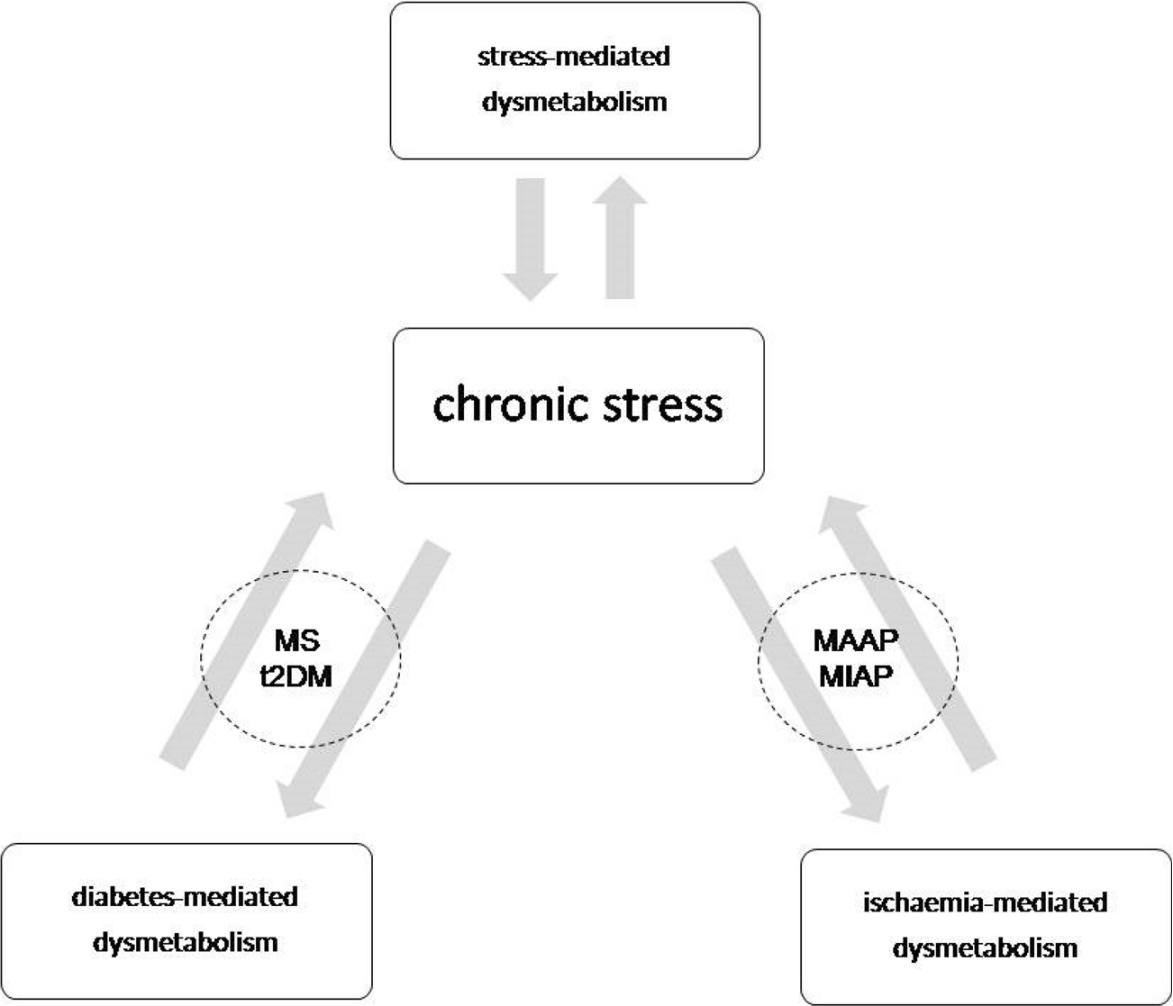
Interrelation between dysmetabolic states. Abbreviations: MS-metabolic syndrome; t2DM-type 2 diabetes; MAAP-macroangiopathy; MIAP-microangiopathy

In summary, the foregoing discussion indicates that in chronic stress the evolution of diabetic dysmetabolism, ischaemia-mediated metabolic disorder, and chronic stress-mediated metabolic dysregulation cannot be separated; each of these can be referred to both as a cause and as a consequence (Fig. 2). The whole process results in a vicious cycle in which chronic stress occupies the central position [3, 12, 14, 48].

Based on the arguments presented above, it can be proposed that any chronic disease state of a vital organ will inevitably lead to the development of diabetes; in myocardial infarct, chronic heart failure, chronic obstructive pulmonary disease, chronic kidney and liver disease, and polycystic ovary disease, the increased prevalence of diabetes is documented [3, 14, 48]. The common denominator of its aetiology is chronic stress (Fig. 1).

It should be noted that chronic stress reaction can be triggered not only by chronic somatic disease but also by chronic psychomental discomfort, i.e., mental stress [20, 49].

It is also important to note that a wide range of morbidities, including obesity, metabolic syndrome, type 2 diabetes, high blood pressure, Alzheimer’s disease, non-alcoholic fatty liver disease, increased risk of malignancies, etc., may also be associated with the presence of chronic stress [26, 45, 47, 49].

## Conclusions

The main messages of our thesis are as follows (Table 3):

**Table 3:** Main conclusions

Chronic hyperglycaemia is a marker and a maker of dysmetabolism	
Normoglycaemia can only be considered as etiological intervention in dysmetabolism caused by insulin deficiency	
Short-term hyperglycaemia is not always a marker of dysmetabolism	
Euglycaemia is not always a marker of eumetabolism	
Metabolic states of both the same organ and the various organs can be different at identical blood glucose values	
Identical metabolic performances can be supported by different biochemical mechanisms associated with identical blood glucose values	
Insulin administration can improve metabolic disorders of various origin	

Chronic hyperglycaemia acts simultaneously both as a marker and as a maker (a trigger) of dysmetabolism; therefore, sustained reduction of hyperglycaemia, i.e., achieving the near-normoglycaemic state, remains the basic therapeutic goal in diabetes treatment and in the prevention of diabetic complications [50].

Although achieving normoglycaemia is a therapeutic target in diabetes, it can be considered as an aetiological approach only in dysmetabolism caused by insulin deficiency.

Short-term hyperglycaemia does not necessarily indicate dysmetabolism.

Euglycaemia is not always associated with eumetabolism.

The metabolic states within an individual organ under conditions with identical blood glucose values can be different: both positive and negative energy balance of cell metabolism can also occur.

The metabolic states of different organs in the organism under conditions with identical blood glucose values can be different: both positive and negative metabolic balance of cell metabolism can also occur.

Identical metabolic performances can be supported by different biochemical energy- producing mechanisms associated with identical blood glucose values.

Administration of insulin, the most powerful metabolic promoter, can improve the metabolic disorders (dysmetabolisms) of various origins. For example, the administration of glucose insulin potassium solution in sepsis has been shown to beneficially influence both clinical outcome and morphological derangements of mitochondria in human liver cells [51].

It can be concluded that the paradigm that blood glucose value is a general metabolic parameter can only be accepted with marked limitations. This statement is justified as follows: blood glucose level is a transport parameter that reflects the actual equilibrium between glucose transport to the blood from the intestinal tract and glucose deposits, on the one hand, and glucose transport into peripheral tissues (glucose disposal) from the blood, on the other hand. For this reason, blood glucose values cannot provide real qualitative and quantitative information about the characteristics of cell metabolism in different organs.
